# The feasibility and impact of deploying a four-tests panel at antenatal care in primary health care facilities of a developing country, Kenya

**DOI:** 10.3389/fpubh.2024.1399612

**Published:** 2024-11-25

**Authors:** Missiani Ochwoto, Micah Matiang’i, Noah Machuki Onchieku, Simon Ndoria, Lydia Matoke, Maureen Otinga, Jeremiah Zablon, Evans Mathebula, Damaris Matoke-Muhia

**Affiliations:** ^1^Innovation Technology Transfer Division, Kenya Medical Research Institute (KEMRI), Nairobi, Kenya; ^2^School of Medical Sciences, AMREF International University (AMIU), Nairobi, Kenya; ^3^Centre for Biotechnology Research Development (CBRD), Kenya Medical Research Institute (KEMRI), Nairobi, Kenya; ^4^Laboratory Medicine and Pathology Department, Brown University, Providence, RI, United States; ^5^Abbott Rapid Diagnostics, Abbott, Woodmead, South Africa; ^6^School of Health Systems and Public Health, Faculty of Health Science, University of Pretoria, Pretoria, South Africa

**Keywords:** triple elimination, antenatal care, cost benefit analysis, test panels, P24 antigen, HIV test, hepatitis B virus infection (HBV), congenital syphilis

## Abstract

**Introduction:**

Contracting HIV, syphilis, hepatitis B virus (HBV), and malaria during pregnancy significantly affects the health of the woman, the pregnancy, and the unborn child. The World Health Organization (WHO) recommends testing pregnant women for these infections to achieve triple elimination of mother-to-child transmissions. However, this goal has not been fully realized in low- to medium-income countries, primarily due to segmented testing practices. This study aimed to investigate the effect of introducing a four-tests panel on the quality of antenatal care (ANC) among pregnant women attending selected Primary Health Care facilities in Kenya.

**Methods:**

Using a multi-design approach, we analyzed ANC medical records from 577 pregnant women attending eight facilities across four different counties. Blood from the women fingerpick was tested for HIV, Syphilis Hepatitis B Virus and Malaria using the four-tests panel and the results compared to those in the medical records.

**Results:**

Out of 577 ANC women, only 8.3% had test results for all four infections available. The majority of the mothers had been tested for syphilis (93.7%), HIV (78.5%), and malaria (62.6%), only 19.5% had been tested for HBV. Testing the women using the 4-tests panel yielded positivity rates of 6.9% for HIV, 0.9% for syphilis, 1.9% for malaria, and 1.1% for HBV. Among those without previous test results, the positivity rate was 2.8% for syphilis, 13.8% for HIV (with 10.6% testing positive for recent p24 infections, F = 24.876, *p* < 0.001), 2.3% for malaria, and 4.5% for HBV, with 83.3% of these individuals having no prior test results. The mean positivity rate of those tested using the 4-tests panel compared to segmented single tests was significantly different. The panel was cost-effective and user-friendly for healthcare workers, and in facilities facing staff shortages, it reduced turnaround time and workloads by half. The use of the panel also improved the profiling of ANC mothers and enhanced data management for the four infections by 91.7%.

**Conclusion:**

Adopting the 4-tests panel has the potential to improve test result outputs, enhance the quality-of-service delivery, and contribute significantly to the achievement of triple elimination goals.

## 1 Introduction

Contracting certain infectious diseases during pregnancy can lead to the death of the mother, fetus, or neonate, as well as other adverse sequelae (1, 2). The World Health Organization (WHO) recommends regular testing and treatment for certain specific infectious diseases among women attending antenatal care (ANC) (3).

Early diagnosis is crucial as it enables timely treatment initiation, facilitates optimal care and management, and serves as a cornerstone of prevention of mother-to-child transmission (PMTCT) programs. Screening protocols for infectious diseases vary based on the regional prevalence of these conditions. In most African countries, women attending ANC are required to be tested for the presence of Human Immunodeficiency Virus (HIV) antibodies, syphilis, hepatitis B virus surface antigen (HBsAg), and malaria parasites (3). In Kenya, the national HIV prevalence among adults (16–65 years) is 4.9%, with a higher prevalence of 6.6% among women of reproductive age (4). While national prevalence estimates for syphilis are lacking, the prevalence among women attending ANC in western Kenya is 3.1% (5), and the WHO estimates a general prevalence of 1.5% (27).

An expectant woman infected with HIV, syphilis, and hepatitis can transmit the infection to her fetus during pregnancy, at birth, or after birth if preventive measures are not taken (7). These transmissions can have a major health impact on the baby, including living with HIV and chronic liver disease in the case of HIV and hepatitis, respectively (8, 9). Babies born with congenital syphilis can suffer from serious birth defects such as blindness, deafness, and anemia and may ultimately face death (10). In contrast, malaria infection during pregnancy can lead to anemia, hemorrhage, maternal death, abortion, stillbirth, low birth weight, infant prematurity, and death (11).

In Kenya, tests for HIV and syphilis are mandatory for women receiving ANC services (12). Tests for malaria infections are administered in malaria-endemic areas, mainly Kenyan highlands, lake regions, and coastal strips (13, 14). Testing for hepatitis B has not been rolled out or mandated for ANC in Kenya, however, there are unreported pockets of HBsAg testing occasionally available in private health facilities.

Although there are protocols that guide infectious disease testing at ANC, their implementation has been marred by challenges in low- and middle-income countries (LMIC). The challenges include low accessibility of quality testing kits, lack of essential kit reagents or machines, poor-quality kits, and low technical capacity of healthcare workers (HCWs) (15). In health facilities, poor infrastructure, lack of storage facilities, and low manpower have posed serious challenges to the implementation of the guidelines (16, 17).

Similarly, issues related to ANC women, such as low or inconsistent turnout for expectant mothers for ANC appointments, perceptions, and cultural beliefs, hinder the implementation of the guidelines (18). A combination of different test kits has been shown to reduce fallout during testing and increase the efficiency of test outcomes. For example, previous meta-analyses demonstrated that dual HIV and syphilis testing prevented adverse disease outcomes and was cost-effective compared to single testing (19–21). Moreover, a multi-country study has shown that combining multiple test kits is not only cost-effective in subsequent visits (22–24) but also increases the rates of testing other infectious diseases. For instance, in Western Kenya, testing syphilis infection increased from 4 to 95% when the test was combined with an HIV routine test (5).

Similarly, in Brazil, the quality, results notification and data management for syphilis infection improved when the syphilis test was integrated into a digital management platform (25, 26).

Most recently, WHO adopted a triple elimination strategy that aimed at improving the test outcome for HIV, syphilis (congenital and maternal), and hepatitis B infection for ANC women (3). Kenya is among the sub-Saharan African countries committed to the WHO’s global strategy to eliminate mother-to-child transmission (eMTCT) of these infections (27). However, this is yet to be realized in most health facilities, mostly because pregnant women are subjected to segmented rapid tests for each infection.

In this study, we investigated the effect of four ANC profiling panels on the quality of ANC among pregnant women in eight selected health facilities in Kenya. We aimed to assess the effect of deploying these 4-test ANC panel tests on routine ANC processes and outputs, evaluate the end-users’ perceptions of the use of the 4-ANC panel, and conduct a cost–benefit analysis (CBA) for deploying the ANC panels in an LMIC. Our findings show that the 4-test ANC panel test improves test result outputs and quality of service delivery, provides value for money, and improves data management processes.

## 2 Methods

### 2.1 Study population and sites

**Figure 1:**
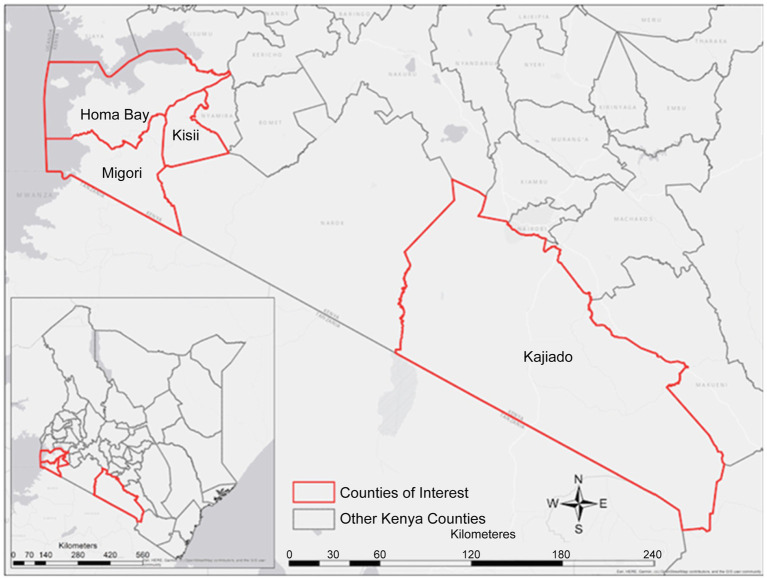
A map of Kenya showing the counties where the study was conducted.

This study was conducted in four counties in Kenya, namely Kajiado, Homabay, Kisii, and Migori (Figure 1). These counties were purposely selected from a list of 15 Ministry of Health (MOH), United Nations Fund for Population Activities (UNFPA) and African Medical and Research Foundation (AMREF) priority counties providing Maternal, Newborn and Child Health (MNCH) services (28). The selection criteria were mainly based on the prevalence of the four infectious diseases in the county and facilities with high volumes of pregnant women from urban and rural settings. Available data shows that malaria prevalence in Homabay and Migori stands at 10–15% (13) [Kenya], hepatitis B virus is 3.5% (4), and HIV is at 19.6 and 13.0% in Homabay and Migori, respectively. In contrast, Kisii County is considered a moderate zone, while Kajiado County is a low zone for the four diseases. The prevalence of HIV and malaria in Kajiado is <2% (4, 13). Although data on the prevalence of syphilis WHO estimated it to be 1.5% (27).

In each county, the research targeted expectant women attending two selected ANC health facilities. The facilities were randomly selected at the county level based on the following criteria: (1) the type of facility (primary or secondary healthcare), (2) the location of the facility (rural or urban), (3) the number of ANC visits per month, with a minimum of 150 mothers, and (4) a reported higher prevalence of any of the diseases of interest (HIV, hepatitis B, and malaria) in that region. Based on these criteria, the following eight facilities were selected: Matasia and Namanga Health facilities in Kajiado County, Marindi Health Center and Magunga Level 4 Hospital in Homabay County, Nduru Sub-County Hospital and Kegogi Health Center in Kisii County, and Ntimaru Sub-County Hospital and Tisinye Health Center in Migori County (Table 1).

**Table 1:** Number of ANC mothers, nurses, and lab techs who participated in the study from each county and facilities.

County	Facility name	ANC mothers			HCW			
		No. of participants (*n*)	Percent (%)	Total	No. of nurses (*n*)	No. of lab techs (*n*)	Percent (%)	Total
Migori	Ntimaru SCH	94	16.3	180	12	1	13.7	20
	Tisinye HC	86	14.9		6	1	7.4	
Homabay	Magunga level 4 Hospital	75	13.0	149	4	1	5.2	16
	Marindi HC	74	12.8		8	3	11.6	
Kajiado	Matasia HC	70	12.1	143	7	1	8.4	24
	Namanga HC	73	12.5		12	4	16.8	
Kisii	Kegogi HC	44	7.8	105	4	1	5.2	35
	Nduru SCH	61	10.6		23	7	31.6	
	**Total**	**577**	**100**	**577**	**76**	**19**	**100**	**95**

HCW = health care worker. ANC = antenatal care. *n* = number, SCH = Subcounty Hospital, HC = Health Center.

### 2.2 Study design

This study employed a multi-design approach: (a) A cross-sectional study design where ANC women coming for a second or more visit were consented to by a trained study enumerator who also assisted them in filling out a digital questionnaire, (b) an experimental design where trained personnel finger-pricked blood samples to test for the four infections, (c) review of medical records of the expectant mothers for tests performed and any laboratory test referral made in the previous visits, (d) focused group discussion (FGDs) and key informant interviews (KII) as qualitative methods used to investigate the perception and uptake of the kits. The purpose and objectives of the study were explained to the expectant mothers in either English or *Swahili* (local language) before requesting consent. The expectant women were also informed that participation was voluntary and that they could withdraw at any point. The study was approved by the AMREF Ethical Review Committee (protocol #: P1101-2021) and all four counties’ departments of health services.

### 2.3 Participant inclusion and exclusion criteria

This study recruited expectant mothers of all ages, irrespective of parity or gravidity, who were attending their second or more ANC visits at the selected health facilities and were willing to provide informed consent and fill out the digital questionnaire.

Women who sought ANC services at the selected facilities for their first ANC visit and had not undergone ANC profile testing at any other facility for the current pregnancy were excluded from the study. The study also excluded women who were referred from other health facilities and whose medical records were not available at the facility or those not willing to consent to participate in the study and complete the questionnaire. To support the project, the study selected at least three HCWs (two nurses and one laboratory technologist) who routinely attended to pregnant women at ANC and laboratory and medical records units.

### 2.4 Assessment of how the deployment of the 4-test ANC panel affects the routine ANC processes and outputs in the selected health facilities

**Figure 2:**
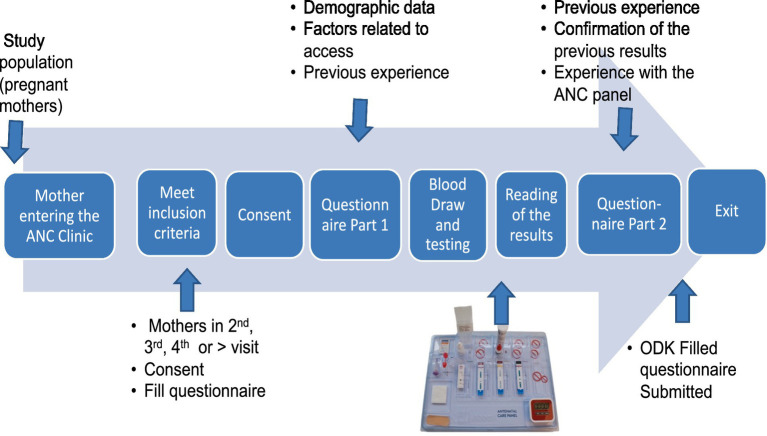
Summary of the entire process from entry to the time the mother exits the ANC facility.

The mothers who were included in the study completed a questionnaire that documented their sociodemographic data and their recollection of previous testing experiences at the facility. The mothers were then tested using the 4-test ANC panel by trained health workers. Since the expectant mothers were at least on their second visit, it was assumed that they had been tested during their previous visit and would recall what tests had been performed. Any positive result for infection was confirmed using the standard conventional kit available at the facility or referred elsewhere for confirmation. Confirmatory tests were conducted according to the national algorithm for HIV and syphilis. The results of the tests were shared with the mothers, who were then asked to complete the second part of the questionnaire, providing feedback on their experience with the 4-test ANC panel. Using the mother’s ANC clinic booklet or card number, the recorded previous test results were documented in the questionnaire alongside the results of the 4-test ANC panel (Figure 2). Disease management was carried out based on the national algorithm.

The sociodemographic data collected included age, parity, gravidity, marital status, level of education, employment status, distance traveled to the facility, and source of financial support for her ANC services, including travel costs. The data collected on their previous experience included whether any test was conducted when they previously visited the facility, enumeration of the tests conducted at the facility, how long it took them to get the test results, and any referral, as well as their general assessment for the previous testing process. We also asked post-testing questions such as their experience with the 4-test ANC panel, how long it took them to get the results, and their general comments for the 4-test ANC panel compared to their previous segmented tests. The flow of activities at the ANC from the mother arriving at the clinic until she left is summarized in Figure 2.

### 2.5 The 4-test ANC panel

**Figure 3:**
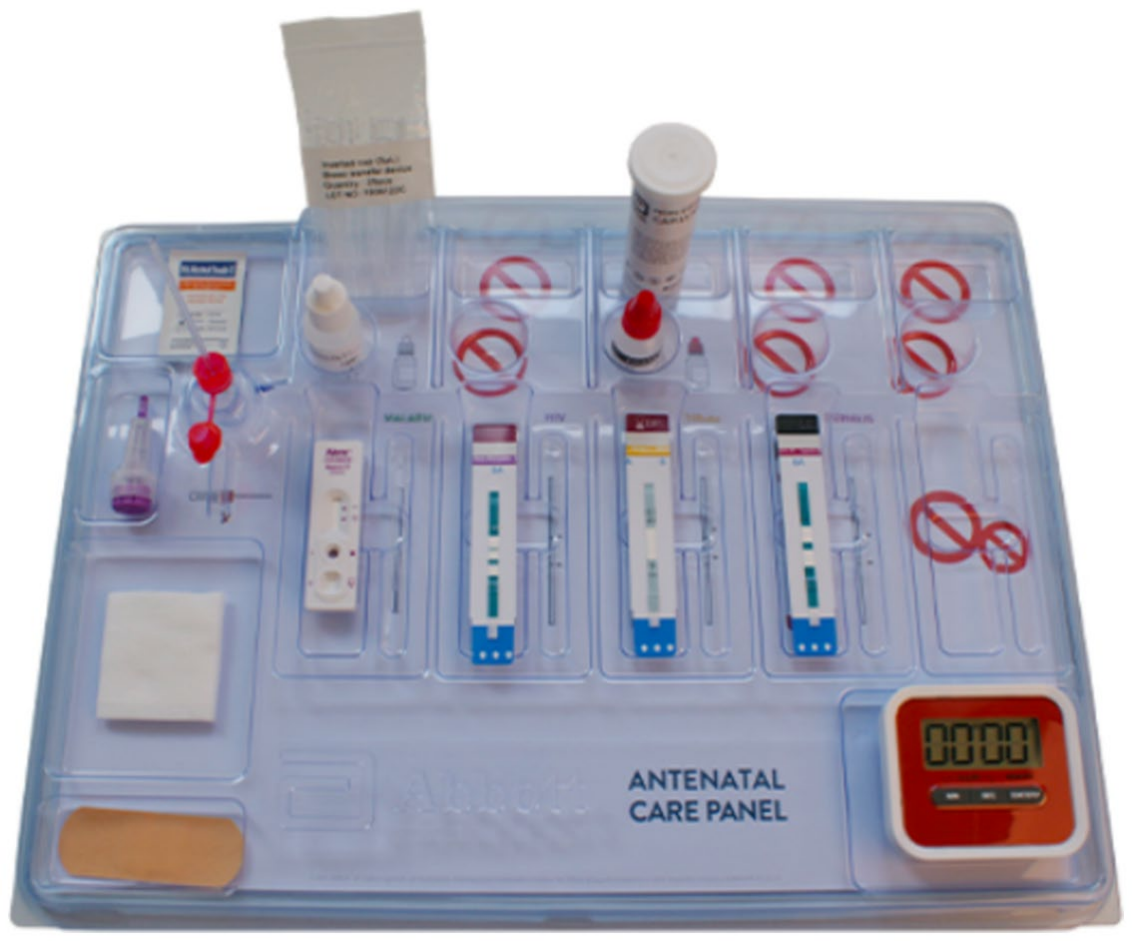
Abbott’s four tests Antenatal Care (ANC) panel, which bundles four crucial diagnostic tests into one panel that requires a single fingerstick of blood for diagnosis. The timer is not included as part of the panel.

The 4-test ANC panel was obtained from Abbott Rapid Diagnostics Ltd. (South Africa). These tests were previously segregated or segmented rapid diagnostic point-of-care strips, which Abbott repackaged into one pouch (also referred to as a panel) (Figure 3). All the tests used fresh whole blood samples from one fingerprick collected with a sample collection tool. The panel test was performed according to the manufacturer’s instructions on a work tray accessory provided. Each pouch contained an EDTA blood sample collection tube, a safety lancet, an alcohol swab, gauze, a plaster, a sample holding tray, and lateral flow immunochromatographic test strips for HIV (*Determine™ HIV Early Detect*), hepatitis B virus (*Determine™ HBsAg 2*), syphilis (*Determine™ Syphilis TP*), and malaria (*NxTek^™^ Eliminate Malaria Pf*) (Figure 3). The sensitivity and specificity of these Abbott test kits had been documented previously and were WHO Prequalified (WHO PQ) (29). A population-specific field evaluation of the test kits was undertaken on a subset (5%) of the total negative samples tested to confirm the specificity in the hands of the healthcare workers in this study. The samples were subjected to the conventional confirmatory diagnostic tests for the four targeted diseases for comparison, and any discrepancy was subjected to further retesting of the sample using ELISA or PCR methods.

### 2.6 Assessment of end-users’ perceptions of the 4-test ANC panel

Front-line ANC HCWs who regularly attended to expectant mothers and who had been conducting tests and/or counseling were trained on how to use the panel. The study PIs randomly visited the facilities to supervise the testing process. This was achieved by observing whether the front-line HCW adhered to the testing procedure and the panel’s processes. The supervisor scored on how the tester correctly utilized the tools provided and skills learned during the training using a scale from 1 to 5, with 1 being low adherence and 5 being full adherence. These include how the tester engaged the ANC mother, how they arranged and utilized the reagents, kit apparatus, and other domains of learning that may affect the resulting outcome.

These aspects were also explored qualitatively through in-depth interviews with mothers and HCWs to assess their acceptance, competencies, and perceptions of the new 4-test ANC technology. Each in-depth interview involved six to eight HCWs or mothers, with one study enumerator leading the discussion while another one took notes. For ANC mothers who had previously undergone testing for HIV, HBV, syphilis, and malaria, their perceptions before and after testing using the new 4-test ANC panel were initially captured through a questionnaire.

The facility’s perception was assessed during in-depth interviews and key informant interviews, which were led by two study enumerators—a discussion leader and a notetaker. These interviews focused on several key issues, including testing disparities, time taken to relay results, the number of women tested by the facility, the cost of test kits, test kit supply shortages, test referrals (if any), tests regularly performed at ANC, and potential effects the new test kit might have if introduced into their facility.

### 2.7 Cost–benefit analysis for deploying the 4-test ANC panel technology in a developing country context

The cost–benefit analysis considered all financial aspects from when the kit was procured to when the ANC mother was tested and left the health facility.

The financial aspects were compared to the conventional segregated tests that are conducted within the facility. The entire CBA mapped out the cost of procuring the testing kits, the estimated cost to run a single sample at the facility that includes the consumables and overhead costs, the cost an ANC mother incurs in sample collection and obtaining the test results, and lastly the cost of referring any mother who was not tested at the facility. The cost of procuring four segregated tests was obtained through KII, FGD, and requisition forms for their supply agencies or other medical records. The cost of the 4-test ANC panel was calculated based on the manufacturer’s supply agent.

A structured questionnaire was developed by a health economist to obtain the cost of running a single test at the facility. The costs were categorized as direct or indirect costs. They included time taken to order and receive each segregated single test kit, time taken to do each segregated single test kit compared to the 4-test ANC panel, resources for conducting single tests compared to the 4-test ANC panel, costs of training, work or backload samples, referrals, recording of data, health communication, and general clinic flow from when the mother starts testing at the laboratory to the time they receive results. All costs related to the tests were accounted for at the health facility level, whether paid for or subsidized from different sources like county government, donors, and health insurance (NHIF and *Linda Mama*). Apart from the direct payments for the tests, mothers bore indirect costs such as time off work and travel costs. The total cost of implementing the four segregated tests was compared to the total cost of implementing the combined 4-test ANC panel using a cost–benefit analysis (CBA).

### 2.8 Data management and analysis

#### 2.8.1 Quantitative data analysis

The Open Data Kit (ODK) application on Androids was used to collect quantitative data, which was transmitted to AMREF servers from the HCW’s mobile phones. The de-identified data was downloaded by PIs and checked for completeness and consistency. Any errors and omissions were communicated back to the field enumerators. The complete data was downloaded from ODK to Statistical Package for Social Sciences (SPSS) version 23 for coding and analysis. On the other hand, descriptive statistics (univariate and bi-variate analysis) were used to derive means, percentages, and frequencies of various variables of interest to investigate the effect of deploying the 4-test ANC panel kits at ANCs in primary health care settings of developing countries. A *p*-value of 0.05 or less was considered statistically significant.

#### 2.8.2 Qualitative data analysis

In-depth interviews and KII (notes and tape recording) were transcribed verbatim and translated to English where appropriate in readiness for analysis. The qualitative data were coded and analyzed using NVIVO 12 software and neural network analysis to identify emerging themes and typologies.

A cost–benefit analysis was conducted by an AMREF financial expert, incorporating both monetary and non-monetary aspects of the project. The analysis utilized evidence based financial frameworks to compare the cost-effectiveness of the 4-test ANC panel against segmented, unilateral testing methods.

## 3 Results

### 3.1 Study participants and HCW characteristics

The study recruited a total of 577 ANC mothers who visited the eight selected study facilities in the four counties from September 2022 to April 2023.

A majority of expectant mothers (89.1%) attending ANC clinics had secondary education as their highest level of education. The mean age was 26.5 ± 5.54 years. A total of 89.4% were married, 84.4% were unemployed, and 23.2 and 31.5% were in parity 0, and 1, respectively. Approximately 4.3% of the mothers reported stillbirths. The cost of the mothers’ ANC services was met by partners (67.1%) or parents (25.0%), and the majority of male partners (87.7%) never accompany mothers to the ANC clinic. The study found that the health facilities predominantly served the local community, with 94.3% of mothers coming from within 10 km, and only 5.4 and 0.3% traveling more than 10 km and 20 km, respectively. The sociodemographic characteristics of the mothers are shown in Table 2.

**Table 2:** General socio-demographic characteristics of the expectant mothers from each county and facility.

Variable: mean (SD)	Categories	Number	(%)
Age: 26.5 (5.54)	16–19 years	36	(6.2)
	20–24 years	213	(36.9)
	25–29 years	169	29.3
	30–34 years	97	16.8
	35–39 years	51	8.8
	40–44 years	11	1.9
Parity: 1.72 (1.57)	0	134	23.2
	1	182	31.5
	2	108	18.7
	3	75	13.0
	4	43	7.5
	5	19	3.3
	6>	16	2.7
Still births: 0.1(0.49)	0	542	93.9
	1	25	4.3
	2	6	1
	3	2	0.3
	5>	2	0.4
Gravidity: 2.78 (1.643)	1	135	23.4
	2	170	29.5
	3	111	19.2
	4	80	13.9
	5	43	7.5
	6>	38	6.5
Marital status	Married	516	89.4
	Single (never married)	56	9.7
	Once married	5	0.9
Employment (source of income)	Not- employed	487	84.4
	Employed	90	15.6
Distance traveled to the facility	< 5 km	319	55.3
	5-10 km	225	39.0
	11-20 km	31	5.4
	> 21 km	2	0.3
Who meets the costs of her ANC services, including travel costs to the facility?	Male partner	387	67.1
	Self	144	25.0
	Parents	30	5.2
	Linda mama	5	0.9
	Insurance	4	0.7
	Both partners	3	0.5
	Not specified	4	0.7

Approximately three-quarters (75.2%) of the participants were selected from counties that are endemic for three infections (malaria, HIV, and hepatitis). The remaining quarter (24.8%) were from low-endemic areas in Kajiado County (Figure 1). The selected participants were from level three healthcare facilities (73.1%), followed by level two (26.9%) (Table 1). All selected facilities were public and located in rural areas with limited access to health services. The number of HCWs working in these hospitals was low. Most facilities had less than 10 nurses and/or midwives and only one laboratory technician in the whole facility (Table 1). Although level four facilities had more nurses/midwives than level 3 facilities, there was no difference in the number of laboratory technicians (Table 1). In this study, a total of 30 HCWs (ANC nurses and laboratory technicians) were selected and trained on testing procedures. Three nurses/midwives and one laboratory technician were selected from each facility.

### 3.2 How the deployment of the 4-test ANC panel affected outputs in the selected health facilities

#### 3.2.1 Previous ANC testing at the facility

A review of the mothers’ ANC booklets (mother and child health booklets) by the HCW indicated that most (99.3%) were tested for at least one of the four infectious diseases at the facility during their previous visit. Approximately 0.7% had not undergone any tests in their previous visits (data not shown). Furthermore, the majority of the mothers (95.6%) indicated that the test(s) was conducted at the same facility, while 4.3% of the mothers had their tests in a different facility. Data from FGD with HCWs revealed that the test kits used in all the facilities were point-of-care rapid tests for each of the four infectious diseases. The HCWs indicated that they were trained before conducting the test at the facility.

Out of 99.3% who had at least one test during their previous visit, 46.8% had received results for two infections, 33.4% for three, and 9.7% for one infection. Only 8.3% had results for all four infections.

**Figure 4:**
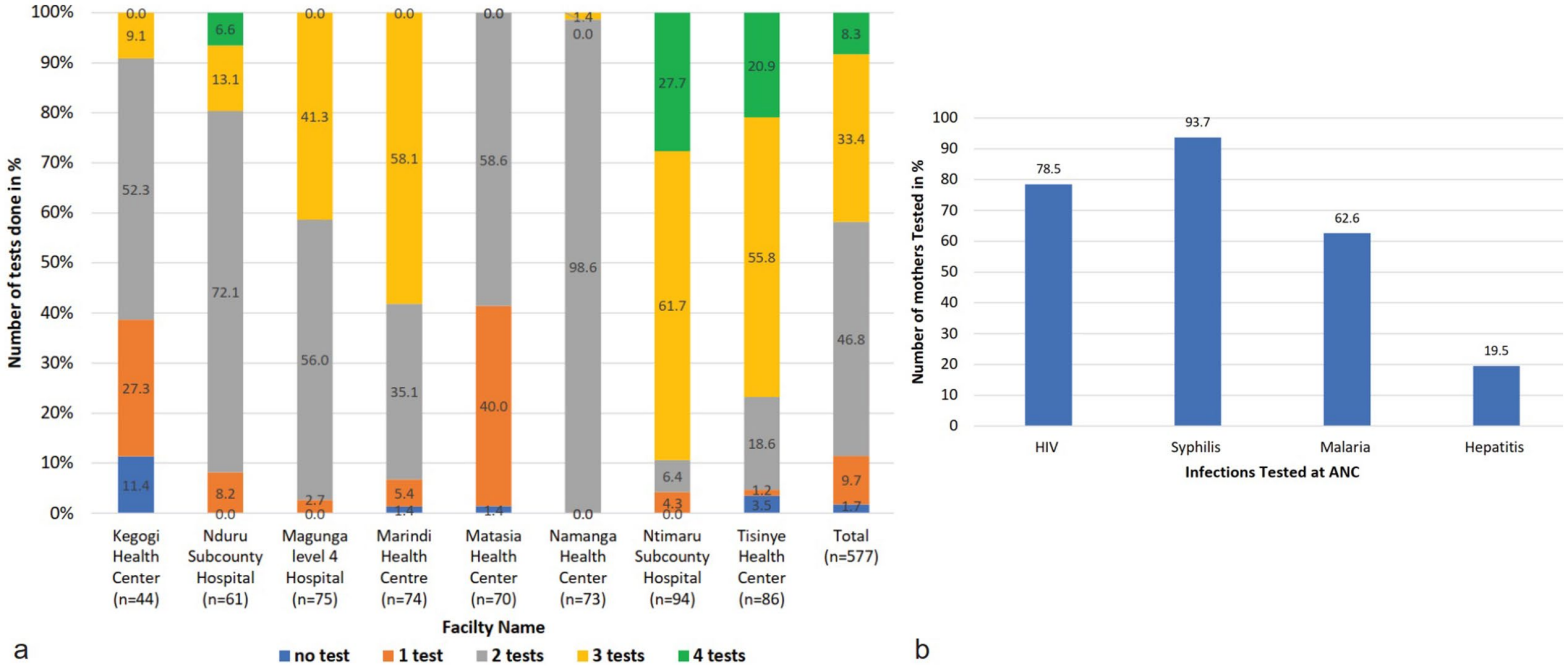
Tests conducted at the facilities **(a)** shows the number of tests conducted at the facilities. Most facilities do two tests, that is, HIV and syphilis tests. Some facilities do three tests that are HIV, Syphilis, and mostly malaria tests. Some facilities performed all four tests. Those facilities are Nduru, Tsinye, and Ntimaru. There are facilities that do only one test or no test at all when mothers visit the ANC. **(b)** Shows the proportion of mothers tested for each infection at the facilities. The most tested infections are HIV, syphilis, and malaria in endemic areas. Rarely is hepatitis B tested at the facility.

In addition, 1.7% of the mothers had never undergone testing for any of the four diseases during their previous visit. The study found that even within the same county, mothers attending different facilities received varying proportions of the tests (Figure 4a). An analysis of responses from ANC mothers across all counties, confirmed by entries in their ANC booklets, revealed that 78.5% had HIV test results from their previous ANC visit, 93.7% had results for syphilis, 62.6% of those in malaria-endemic areas had malaria test results, and only 19.5% had been tested for Hepatitis B (Figure 4b). There was no uniformity in the tests administered, regardless of the type of facility or whether the tests were mandatory or recommended.

#### 3.2.2 Testing at the facility using the 4-test ANC panel

**Figure 5:**
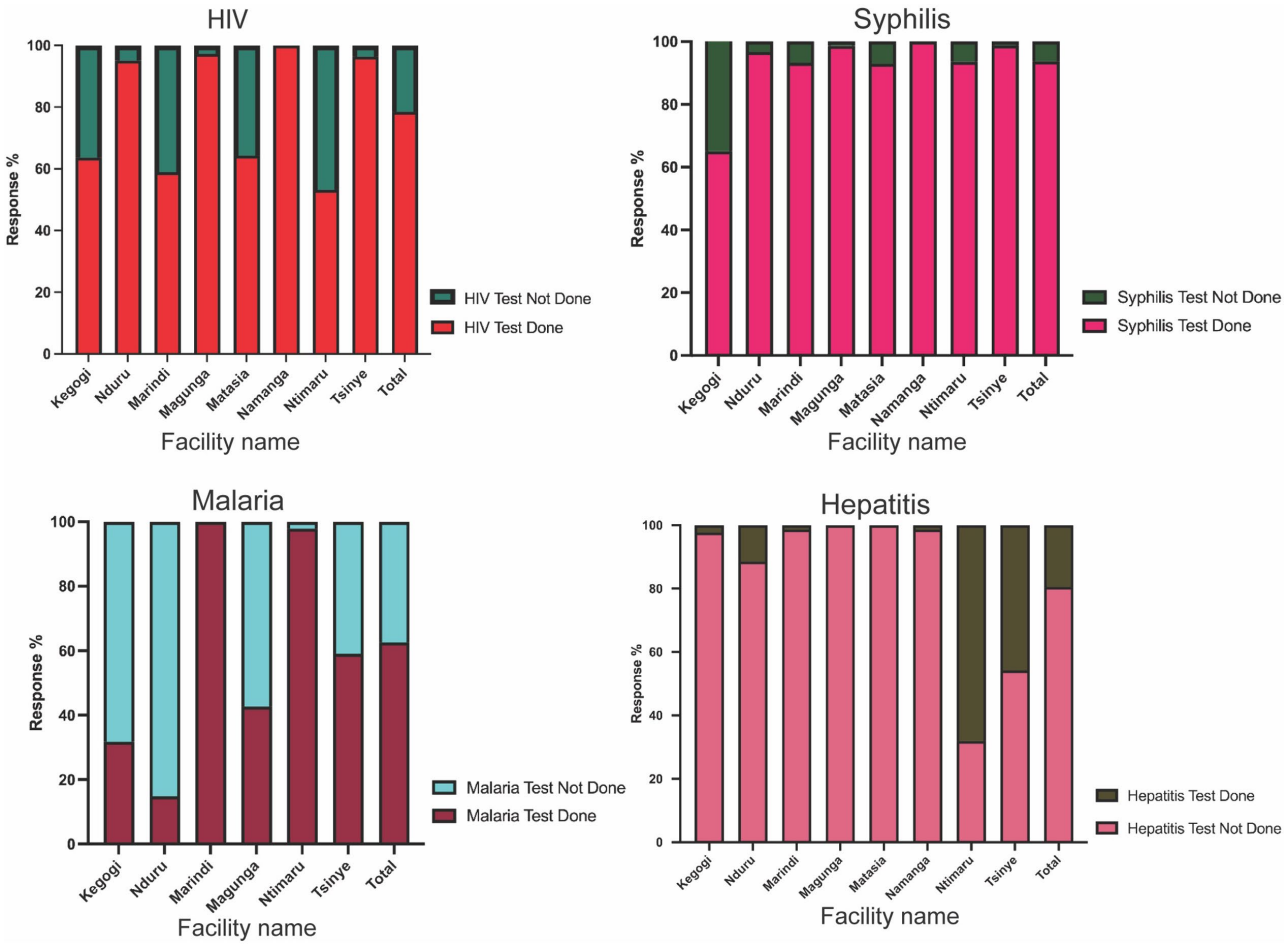
A Proportion of mothers tested for HIV, syphilis, malaria, and hepatitis B virus at their first or previous visit to the ANC facilities. There was no uniformity in the number of tests conducted within the counties. **(a)** The majority of the facilities performed HIV tests. In all facilities, more than half of the mothers had tested for HIV. **(b)** Most facilities test for Syphilis just like HIV, with all facilities testing more than 90.0% **(c)** facilities in Malaria endemic regions tested for malaria. Only two facilities tested more than 98.0% of the mothers, while the rest of the facilities tested less than half of the ANC mothers. **(d)** Very few facilities test for hepatitis B at the ANC. Only two facilities tested more than half of the mothers. Some facilities never recorded a single test for Hepatitis B.

To adequately address the gaps observed in the routine testing and standardize the process, the study tested all ANC mothers using the 4-test ANC panel tests and compared the results with their previous test results. The data was subjected to an independent t-test, which suggested that there was a statistically significant difference in the mean of the number of tests conducted at the ANC facility using segregated tests and the mean when tested using the 4-test ANC panel for the four infections (*F* = 9.596, *p*-value 0.02). The findings of these tests were categorized per infection (Figure 5).

The data depicted in Figure 5 further revealed that almost 21.5% of the mothers had not undergone HIV testing in their previous visit, despite it being a mandatory ANC test. It was also observed that level four and sub-county hospitals had the highest numbers of mothers who had tested for HIV compared to level three health facilities [Magunga Level 4 Hospital (97.3%), Nduru Subcounty Hospital (95.1%), Namanga Health Center (100%), or Tisinye Health Centre (96.4%) versus Kegogi Health Center (63.6%), Marindi Health Center (58.9%), Matasia Health Center (64.3%), or Ntimaru Subcounty Hospital (53.2%)]. A comparison of the two facilities selected within each county showed that there was no observed consistency, where one facility had a higher proportion of HIV tests compared to the other (Figure 5a). Data from KII and FGDs with the HCWs showed that the availability of test kits was the main reason that underlies the lack of testing of the ANC mothers.

**Figure 6:**
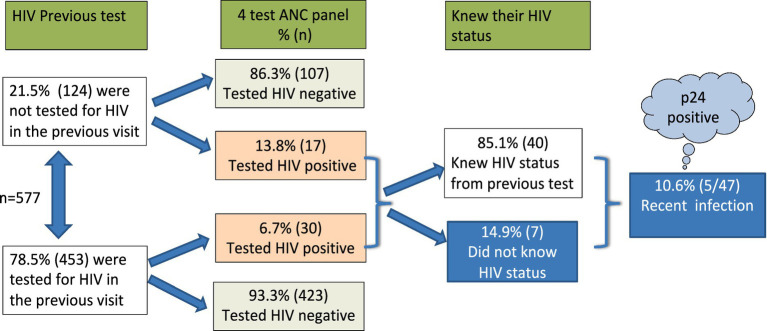
HIV testing schematic diagram using a 4th generation, 4-test ANC panel with an additional component to test for p24 early detection. Recent infection was at 10.6% of the mothers who attended ANC.

To standardize testing disparities, we tested all 577 mothers (regardless of previous HIV test results) for HIV using the 4-test ANC panel and an HIV strip. The overall HIV positivity rate was 8.1% (47/577) among ANC mothers across the four counties under study. All positive test results were confirmed using the current HIV national algorithm. Among the mothers with recorded previous HIV test results, 6.7% (30/453) tested HIV positive. In contrast, 13.8% (17/124) of the mothers who had no prior HIV test record tested positive using the 4-test ANC panel. Further investigations showed that 85.1% (40/47) of all the mothers who tested positive with the 4-test ANC panel were already aware of their HIV-positive status, having been tested previously. However, 14.9% (7/47) were new testers and were completely unaware of their HIV-positive status (Figure 6).

The Abbott 4-test ANC panel for HIV is a fourth-generation test that includes an additional component for early detection of the p24 antigen. Notably, among the HIV-positive individuals, 10.6% (5/47) tested positive for the p24 antigen, indicating a recent infection, and were unaware of their HIV status. Both the t-test and Levene’s test for equality of variances indicated that the variances between the group previously tested for HIV and the group tested using the 4-test ANC panel were significantly different (*F* = 24.876, *p* < 0.001).

#### 3.2.3 Syphilis testing at the facility compared to the 4-test ANC panel results

Syphilis is a mandatory test for women in all ANC facilities in Kenya. Our analysis showed that 93.7% of the women had been tested on their previous visit (Figure 4b). It was observed that health centers and level 4 or sub-county health facilities had tested more than 90% of the mothers attending the ANC clinic, except Kegogi Health Center, which tested 65.1%. Only 36 (6.3%) mothers had never tested for syphilis at the collective ANC facilities (Figure 5b).

When the mothers were tested using the 4-test ANC panel, syphilis positivity was 0.9% among the study population. Among mothers who had not tested for sin their previous visit, only one (2.8%) tested positive, which revealed that 2.8% of syphilis-positive mothers miss their interventions due to lack of testing. On the other hand, the study observed that in every 5 cases positive for syphilis, one case was not tested during the first visit.

#### 3.2.4 Malaria testing at the facility compared to the 4-test ANC panel results

Malaria tests are recommended in facilities located in malaria-endemic regions. In this study, a total of 143 ANC mothers from Isinya and Namanga health facilities, where malaria is not a routine test, were excluded from the analysis. The overall malaria testing rate in the endemic regions was 62.6% (Figure 5c). Although malaria testing is recommended in these regions, it was rarely tested (Figure 5c). KII and FGD revealed that malaria tests that were never conducted were not referred to any other facility.

All ANC mothers in the endemic and non-endemic regions underwent malaria infection tests using the 4-test ANC panel kits. A malaria positivity rate of 2.1% was realized among the study population. Among those who had tested in the previous visit, 1.9% tested positive, while among those who had not tested for malaria in the previous visit, 2.3% tested positive. All the malaria-positive cases were in the malaria-endemic regions (data not shown).

#### 3.2.5 Hepatitis B virus testing at the facility compared to the 4-test ANC panel results

The WHO recommends testing for hepatitis B among mothers attending ANC. In Kenya, hepatitis B testing has not been prioritized in many facilities. When the study mothers were asked if they received hepatitis B tests at their ANC visit, the study found that up to 80.5% had never tested for hepatitis B virus (Figure 5d). Only 19.5% had received hepatitis B tests. Further analysis showed that the majority (93.0%) of those who tested for hepatitis B was an initiative of a partner for Ntimaru sub-county hospital and Tisinye health center, which were in one county-Migori (Figure 5d). Up to five facilities had recorded very low tests for the hepatitis B virus. The FGD revealed that there is very low hepatitis B awareness in these facilities.

The study recorded 0.9% hepatitis B virus positivity. Among those who had tested for Hepatitis B on their previous visit, one participant (0.9%) tested positive for HBsAg. Among the ANC mothers who had not tested for HBsAg in their previous visit, five (1.1%) tested positive using the 4-test ANC panel kits. Generally, in every six HBV-positive patients, only one knows their status.

The distribution of HBV was observed across the four counties with varied burdens. Homabay County accounted for half of the cases. Kajiado had 33.3%, while Migori had 1.7%.

### 3.3 Impact of the 4-test ANC panel on routine ANC processes in the selected health facilities

#### 3.3.1 Availability of all 4-test ANC panel kits

The FGD and KII, along with the HCWs and ANC mothers, revealed several reasons for not doing tests at the facility during their previous visit. The data showed that the unavailability of the 4-test ANC panel kits at the time of the visit was the main reason for the lack of testing for the infections. The ANC mothers and HCWs reported a shortage of these test kits and an unpredictable supply chain. Further, laboratory technicians averred that there was also a shortage of reagents for sample collection:

*“We were not testing for malaria because we sometimes lack the reagents. However, for Hepatitis B, we do not test because we do not have reagents to perform the tests.” –* A laboratory technician reported during the FGD session.

A majority (80.0%) of the HCWs attributed the shortage of reagents and stockout of the kits at the ANC facilities to a defragmented supply chain. One key informant said,

*“…we make orders of reagents from Suppliers, and at times, they do not deliver on time, or we get less than what we have ordered,”* – A key informant said during the KII session.

#### 3.3.2 Referral

If the facilities cannot test the mothers, they are allowed to refer them to other facilities within. This ensured that the mothers did not miss any of the recommended tests. It was observed that the facilities under study referred a small percentage (4.5%) mostly to facilities that were within 5 km (57.7%) or within 10 km (42.3%).

#### 3.3.3 Where and who performs the ANC tests

**Figure 7:**
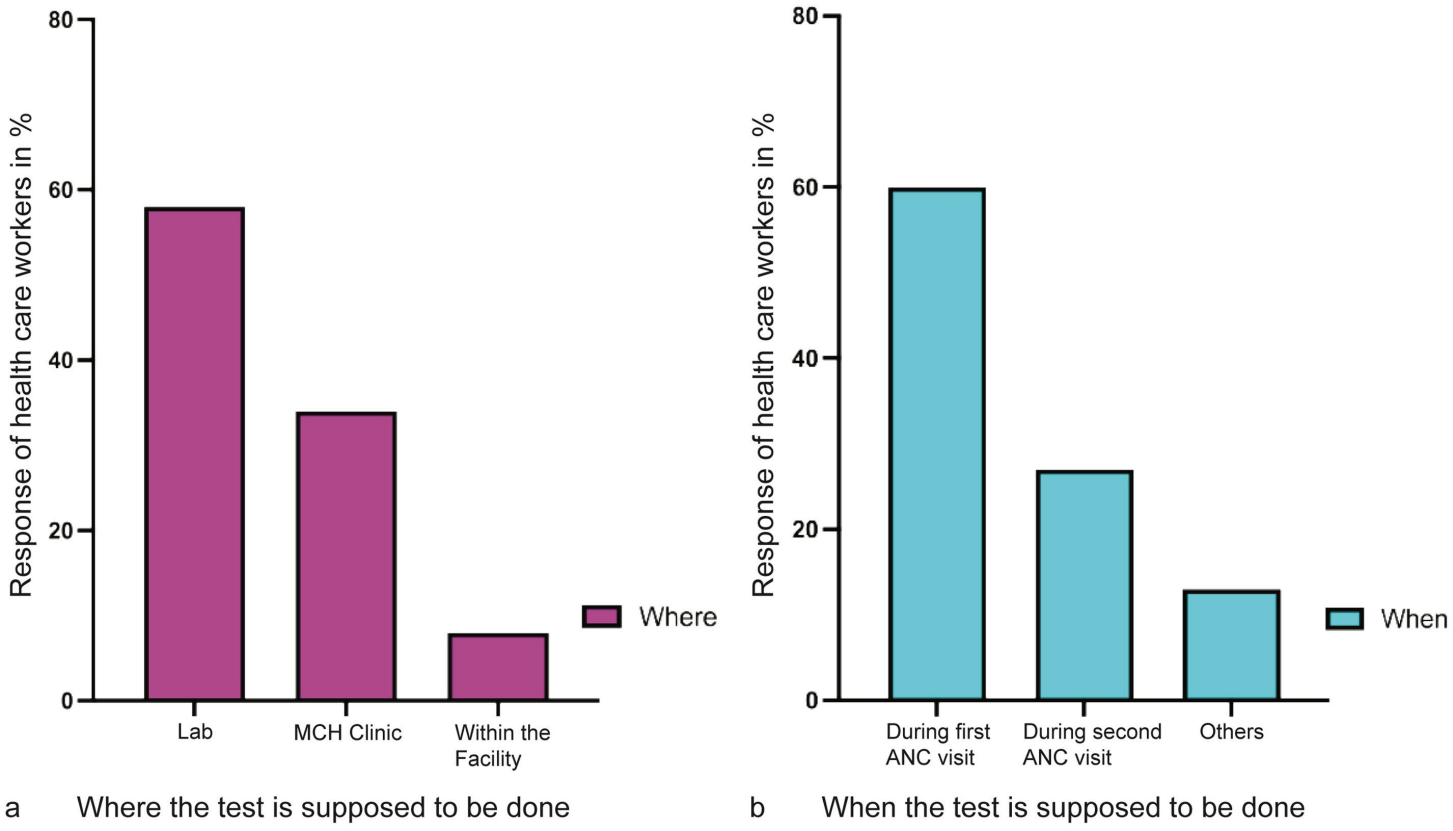
Location where and when the test is supposed to be conducted and during which ANC visit. **(a)** Coded responses of health care workers, the majority responded that the test should be conducted at the laboratory as compared to the MCH clinic. Very few healthcare workers were undecided about where the test would be carried out. **(b)** The majority of the healthcare workers agreed that all tests should be conducted during the first visit. However, 26% had no problem doing the ANC test in the second or any other visits.

Where and who does the test emerged as an area of concern. From the responses during the FGD and KII interviews. It emerged that the mandatory tests (HIV and syphilis) are conducted within the ANC facility by the nurses and the midwives, whereas the other recommended tests (malaria and hepatitis) are conducted at the laboratory. When the HCWs were asked where the first ANC test should be conducted, 58% responded that all tests should be performed in the laboratory, 34% would like the tests to be conducted at ANC, and 8% were indifferent as long as the test is conducted within the facility (Figure 7a). A section of mothers at an FGD believed that these disparities resulted in blame games that delayed testing results.

Similarly, analysis of when the tests should be conducted showed that the majority (60.0%) of the HCWs preferred to have the tests conducted during the first visit. The remaining 40.0% had no problem doing the test any other time of the visit before delivery (Figure 7b).

One respondent, in responding to where and when to do the test, said:

*“…You know a mother can visit the ANC clinic for the first time or visit today. We do some tests at ANC and send her to the laboratory for other tests, which means we will record the two tests, but the other tests (those done in the laboratory) will wait till their next visit. If the mother is infected, the results from the laboratory will not be of much use to her because she will access them in the next visit…”*.

Another key informant added,

*“…Sometimes the laboratory might lack test kit or reagents for a given test, which means the mother will miss that test in her first visit, which can happen again in the second and any other visit… Eventually, this will affect the mother’s access to care. The mother will blame us, but we have nothing to do.” –* Key informant.

#### 3.3.4 Staffing and workloads

Staffing was equally highlighted as one of the key challenges facing ANC testing and service delivery. Most of the key informants and respondents confirmed that since they are competent and the facility might be equipped to perform the tests, a staff shortage is an impediment.

The compounding effect of understaffing in the ANC department of the health facility is far-fetched. One of the immediate impacts is increased workload, as mentioned by the respondents.

“*At times, the workload can be too much because maybe the nurse who is operating the ANC is the one still operating child welfare,” said the speaker during FGD.*

With increased awareness of the uptake of ANC services by pregnant mothers, it is important to appreciate the impact that workload will have on the adoption and scaleup of the 4-panel test kit.

#### 3.3.5 Data management

Data management was similarly highlighted as a challenge to the ANC testing. During the FGD session, missing data was one of the major issues that was raised.

*I think sometimes we don’t offer the four tests because in the Mother ANC booklet there is no provision to record malaria and hepatitis results, such results are recorded only in the lab. So sometimes there could be missing data at ANC not because it was not done but it was not recorded anywhere at the end” – Speaker during FGD session.*

Therefore, effective ANC interventions should include reliable and consistent data management. Data management, as a prerequisite to evidence-based learning in the ANC sector, must be effectively addressed before the rollout of kit utilization. The staff within the ANC must have the requisite skills, infrastructure, and updated tools to consistently and reliably capture data. They should also have the basic skills to draw insights from the data patterns from the ANC following the adoption of the 4-test ANC panel kits. Data consistency in the ANC tests encompasses data on procuring kits and reagents for the mothers administered with the testing services. There is a need for a comprehensive ANC data management system guided by a holistic roadmap that guides the whole critical data management pathway.

### 3.4 Cost benefits of the 4-test ANC panel

#### 3.4.1 Costs to the health facility

We compared the overall cost of the current standard of care segregated kits to the estimated costs of the 4-test ANC panel kits under study in the cost–benefit analysis. We analyzed our results as costs linked to the health facility, those directly linked to the mother, and any cost benefits to the two parties.

At the warehouse or distributors, a package of 25 units of the 4-test ANC panels costs Ksh. 26,300, translating to Ksh. 1,052 per test set. In comparison, a set of segregated test kits costs Ksh. 877. Health facilities provide estimates of monthly costs for consumables and overheads, including items for sample collection, such as syringes bandages, as well as facility overheads such as electricity/power, testing space, and water bills directly attributable to conducting ANC tests. The total cost of using the 4-test ANC panel was Ksh. 643, which is Ksh. 2,516 less than the cost of four segregated kits, totaling Ksh. 3,160.

Our findings show that the 4-test ANC panel, which is designed to be conducted by a single healthcare worker at the point of care using one sample, costs Ksh. 665, which is 39.0% cheaper than the four segregated tests, which are performed by three different HCWs at a cost of Kes 1,094. The data also showed that 69.0% of the segregated tests are conducted by lab technicians, whereas any healthcare professional can perform the 4-test ANC panel. For a single set of segregated tests, the three categories of HCWs spend a combined 112.5 min, whereas the 4-test ANC panel takes 75 min.

**Figure 8:**
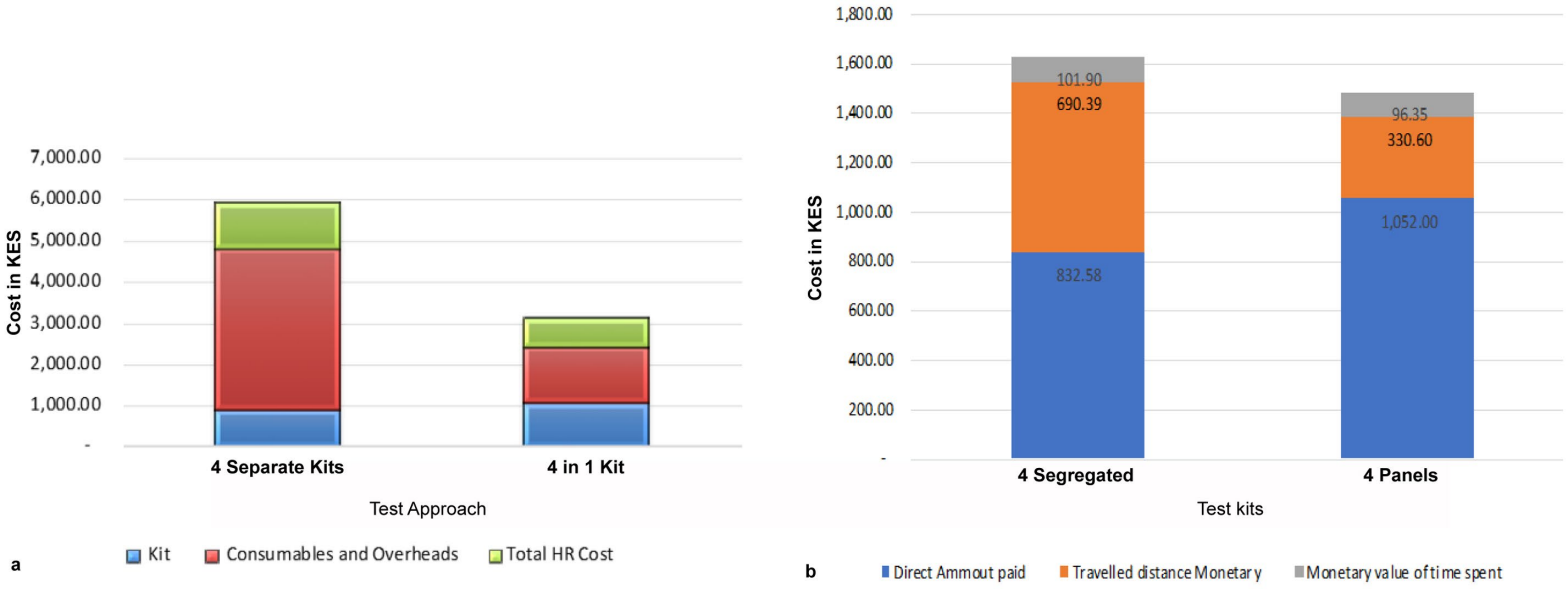
Cost-effectiveness of the 4-test ANC panel. **(a)** Comparison of total costs of delivering a set of the 4-test ANC panel: it costs a health facility Kshs 5,132 to perform the segregated the 4-test ANC panel, whereas it costs Kshs 2,361 to perform the same tests using the 4-test ANC panel. Therefore, a facility can save 54% of the total cost of the tests. Most of the savings are on consumables and, overheads, and human resources. **(b)** The cost incurred by a mother attending ANC. The 4-test ANC panels are cheaper than the segregated tests.

Overall, the cost of performing tests using the 4-test ANC panel is 56.0% lower than the cost of using segregated tests (Figure 8a).

#### 3.4.2 The cost to the mother

The mothers attending ANC also bear direct costs payments for the kits, transportation, waiting time for test results, and any referral expenses. Assuming the cost of the kit is recoverable from the mother, the 4-test ANC panel test is 9.0% cheaper than the four segregated tests. However, the direct costs for the mother were 26.0% higher for the 4-test ANC panel compared to the four segregated tests due to the cost of purchasing the kits. In contrast, the estimated transport cost to the facility for the four separate tests was 52.0% higher than for the 4-test ANC panel, as the mother may need to make an additional visit if a test is missed. Additionally, the cost of time spent waiting for results from the 4-test ANC panel test was 5.0% lower than for the four segregated kits (Figure 8b).

## 4 Discussion

Expectant mothers are encouraged to seek ANC services at 16 weeks to prevent pregnancy complications and vertical infectious transmission (7, 30, 31). Several studies have shown that, in Kenya, expectant mothers presented for their first ANC visit between 21 weeks and 28 weeks (32–34). During these visits, the HCWs test the mothers for recommended infectious diseases that can threaten the health of the mothers and the unborn, among other screens (1, 30). The Kenya PMTCT guidelines recommend HIV and syphilis tests for all expectant mothers and malaria, HBV, and HCV tests in certain populations (23). This study is intended to assess the feasibility and impact of deploying a 4-test ANC panel at ANCs in eight primary healthcare settings.

The study tested and interviewed 577 mothers seeking ANC services at eight rural health facilities across four counties in Kenya. These facilities served ANC mothers within a 10 km radius (94.3%), indicating that most mothers seek ANC services at the nearest healthcare facility. As a result, interventions targeting these facilities directly benefit the local communities. The rural facilities in the study were characterized by a low number of HCWs, including laboratory technicians and nurses or midwives, falling below the WHO recommendation of one nurse per five patients (35).

The available nurses and technicians represent the population that would utilize the 4-test ANC panels in primary healthcare settings.

Despite the staff shortages, it was encouraging to observe that HCWs were able to perform the 4-test ANC panel without any discrepancies. This success was partly due to their prior experience with rapid test kits, as well as the refresher training and supervision sessions conducted before the study rollout. The training sessions helped identify specific needs and significantly enhanced the skills and knowledge of the HCWs. In situations where HCW numbers are low, the use of easy-to-operate devices and technology is highly recommended, and the implementation of such innovations should be accompanied by training and supervision (36).

The introduction of the 4-test ANC panel significantly impacted testing outputs of the four infectious diseases at ANC *(F = 9.596, p-value < 0.02)*. Prior to the introduction of the 4 ANC tests panel, only 8.3 and 33.4% of the mothers were tested for all four and three infections, respectively, leaving most ANC mothers and unborn babies at risk of vertical transmission. The HCWs attributed this low number of tests to the limited availability of test kits and reagents or the lack of national guidelines on some tests, such as hepatitis B. Such gaps at a national level can be barriers to achieving the UNITAID 95–95-95 and WHO triple elimination initiative that encourages countries to simultaneously commit to eliminating mother-to-child transmission of HIV, syphilis, and hepatitis B (15, 27, 37). Although the Kenya Ministry of Health has mandated testing for HIV and syphilis in all ANC facilities countrywide, this study observed that only 78.5 and 93.7% of the mothers had been tested for HIV and syphilis, respectively, which was lower than the national HIV target of 90-90-90 (38). Malaria and hepatitis are recommended tests in counties where the diseases are endemic. However, the number of ANC mothers who were tested in these counties remains low, with malaria at 62.6% and hepatitis at 80.5%. Overall, ANC mothers not tested at ANC clinics were 21.5% for HIV, 6.3% for syphilis, 27.4% for malaria, and 80.5% for HBV. The main reason for not testing was the availability of test kits and reagents. This explained why facilities within the same county had different numbers of mandatory and recommended tests. KII and FDG revealed that if the 4-test ANC panel are adopted, the fragmented and missing tests will be minimized, and the testing gaps will be resolved.

The study noted that using the panels will increase access to quality ANC outcomes. When all the ANC mothers were tested for HIV, the proportion of positive mothers was 8.2%, which is slightly higher than previously recorded in the same region (39). The difference can be attributed to the testing methodology, with the HIV test in this study being a 4th generation and able to detect p24 antigen, an indicator of early infection. It was noted that among the 14.9% of mothers that tested positive for HIV had never tested before, and 10.6% of those positive cases were positive for 24, suggesting early infection. The WHO recommends linking to care any HIV-positive mothers. Without a highly sensitive kit with the ability to detect early infection biomarkers, such a process may be delayed to the disadvantage of the unborn.

HIV infection can occur at any point before pregnancy, during, and after delivery. Expectant mothers attending ANC should know their infection status during pregnancy because of the risks involved. There are reports of expectant women seroconverting during pregnancy, and many reports show that women are sexually active when expectant (40). This raises questions on the frequency at which expectant women should be tested during and postnatally. A repeat test is recommended in the third trimester for pregnant women with initial HIV-negative tests (41, 42). However, the earlier the test is conducted, the better it is for the mother and the newborn. Early detection and initiation of ART were shown to be critical in controlling continued transmission and significantly reduced premature mortality by 11.2% when data from 528,234 individuals was analyzed (43).

The study also revealed that one in every five syphilis-positive cases was not tested, meaning facilities missed 2.8% of syphilis-positive cases among those not tested. This is concerning, as it may suggest that one in five children could develop congenital complications from a curable syphilis infection (10). According to the Ministry of Health (MoH) guidelines, malaria testing is required only in endemic areas. When all ANC mothers were tested for malaria using the 4-test ANC panel kits, no positive malaria cases were detected in counties outside malaria-endemic regions. This finding supports the rationale for segregated malaria testing in the country.

Although pregnant women are at an increased risk of HBV, which is predominantly sexually transmitted, it was the least tested disease among the ANC mothers, with only 19.5% having been tested prior to the study. In Kenya, the burden of Hepatitis B infection in the general population is estimated to be 3.5% (4). In our study, the prevalence among pregnant women across the four counties was 1.1%. Awareness of hepatitis B was low among both mothers and HCWs, which was consistent with findings from Ngaira et al. (44).

Similarly, HBV-infected mothers may vertically transmit the virus to their neonates if the infants are not vaccinated within 24 h of birth. Our findings indicated that 1.1% of positive cases were missed, meaning one in every 100 mothers seeking ANC services was not tested.

The lack of a reporting structure for malaria and hepatitis results, unclear testing guidelines, low awareness, and a lack of test kits were the main reasons the HBV test was not conducted. The 4-test ANC panel has demonstrated the potential to increase the number of expectant mothers being tested, enhance case finding, and reduce missing data by ensuring that all tests are conducted simultaneously.

This approach could be instrumental in implementing the triple elimination of mother-to-child transmission (7, 20, 27). Regarding hepatitis B testing at ANC, the elimination of hepatitis by 2030 and reducing the disease burden will only be achieved by integrating HBV testing with other infections, such as HIV and syphilis. Infants born to infected mothers are at significant risk of transmission if there is no intervention (45), with up to 95% of these infants likely to develop hepatocellular carcinoma (46).

Although the cost of acquiring four separate tests was Ksh. 174 lower than the set of 4-panel kits, factoring in the consumables, overheads, and human resources increased the total cost of the segregated tests by Ksh. 2,516. The 4-test ANC panel kit is designed to be conducted by a single healthcare worker (HCW) at the point of care using one sample, whereas the four segregated tests require at least three HCWs. This approach not only saves time but also reduces the physical space needed, optimizes testing flow within the facility, and cuts overhead costs (47).

The time taken by HCWs to perform each test was calculated, and the value of this time was based on their monthly salaries. This reflects the actual cost to the health facility, which pays the healthcare workers, as well as the opportunity cost of having these workers engaged in testing when they could be attending to other tasks. The data revealed that laboratory technicians conducted 69% of the tests, a resource that was in short supply at the facilities. In fact, five out of eight facilities had only one laboratory technician. By using the 4-test ANC panels, there is potential for task shifting, which would improve human resource management by allowing other HCWs to assist with testing.

In this study, we demonstrated that combining the 4-test ANC panel into a single panel reduces the cost of running the test at the facility more than the cost of acquiring the kit from the supplier. This means quicker ANC testing, efficient early detection and initiation of treatment, improved client-physician relationship, timely service delivery as compared to before, increased retention of mothers, improved trust in the ANC service delivery, reduced referral to the private clinics, positive feedback (happy) from the clients, among others. One participant summarized this observation by saying,

*“I consider it to be a good thing because even if you get a mother who has come for only one visit, in that one visit, you at least will be able to capture those four tests at once. So, even for the mothers who don’t normally come for the ANC visit and do not follow up with the four or five visits, That one visit captures four tests, which is a good thing even if the mother has come late. Those four tests at once, I think, are very good.”* – Speaker at FGD Session.

Although most of the male partners met the cost of ANC services, including transport costs, they were not present at the clinic to make financial decisions. This would affect any increase in cost. Out of the 25.0% of the mothers who cater for their ANC costs, some might arise from decisions made at the facilities without further consultation. In addition, although the insurance and *Linda Mama* programs were not common methods, they proved to be the most reliable and the best methods to meet the ANC costs.

The perception of HCWs and ANC mothers on the use of the 4-test ANC panel was very good, reflected from the time of training to the application of the new 4-test ANC panel. Although it was noted that the concept of rapid test kits (RTK) was not new, the concept of combining them into a single panel was unique. This indicated that even before the 4-test ANC panel training, the HCW at the facility could test almost all ANC mothers attending their facility. In rolling out the 4-test ANC panels, HCWs may not need rigorous training; refresher training will suffice. A similar observation was made when rolling out SARS-COV-2 RTK in Kenya (48). The quality control results performed on positive and negative samples confirmed the capacity of HCWs to correctly test the ANC mothers who visit those facilities. Apart from the laboratory as the main place for conducting the ANC tests, some of the tests, such as HIV and syphilis, were conducted at the MCH for triple elimination of HIV, syphilis, hepatitis, and malaria. Training nurses and other ANC midwives to perform the tests and refer to any complex situation is necessary. Our study demonstrated that such HCWs can perform these tests easily.

## 5 Conclusion and recommendations

1. There is a need to invest in integrated, easy-to-use approaches to increase universal access and achieve triple elimination of HIV, syphilis, hepatitis B, and malaria. The 4-ANC test panel will improve the quality of ANC services offered in the country.
2. The health facilities have the capacity to use point-of-care tests (RTK). This capacity can be utilized to roll out a more robust testing system, like the 4-ANC panel, with minimal training but high-quality outcomes.
3. In the current testing at ANC, there was no uniformity in the tests conducted in all facilities, whether mandatory or recommended. The low numbers of ANC mothers who tested for all four infections (8.3%) can be boosted, synchronized, and integrated if the tests were not segregated. A similar observation was made on individual tests. Of the four tests, syphilis was the most performed at 93.7%, followed by HIV (78.5%), malaria (62.6%), and hepatitis B was the least (19.5%).
4. Health facilities were understaffed, and any lengthy test procedure burdens them. There is a need to centralize the ANC testing and reduce the testing workflow. Using the 4-ANC test panel is the best way to do this. Furthermore, using the panel will reduce the number of referrals observed and the number of missing data in reporting for four infections.
5. By not testing ANC mothers in the first ANC visit, 13.8% who are infected will miss a chance to know their status. This is the barrier to accessing universal health care. Mechanisms should be implemented to ensure there is no missing opportunity to test the mothers at the first ANC visit. Increasing the frequency of testing for infectious diseases improves universal access.
6. HIV infection can occur at any time during pregnancy. This study highly recommends repeating all negative expectant mothers, preferably with a 4th generation kit (p24 test). This study has shown that combining the tests will improve the quality of data collected, consolidate it and promote data completeness for key health players and the decision-making process. This will promote universal access to health care.
7. By using the 4-test ANC panel, the health facilities will save 47.4% of the total cost, HCW will save 69.0% valuable time, minimal lab referrals, and timely results, and the panel is 9.0% more convenient to mothers than segregated tests. We strongly recommend the country and other LMICs embrace the use of the 4-test ANC panel.
8. Data repositories are critical in national programs and data-driven decision-making. WHO has an elaborate strategy for triple elimination of HIV, syphilis, and hepatitis B. However, without data documentation, this dream would not have been possible. This study has demonstrated that collecting data using the 4-test ANC panel test kit will improve the quality of the data collected and fill the gaps resulting from segregated testing. We recommend that LMICs use combined testing and embrace electronic medical records to bridge gaps experienced in generating and managing programmatic data.

### 5.1 Limitations of the study

These findings should be analyzed during the COVID pandemic, where processes were slow; we cannot rule out that the COVID-19 epidemic might have affected the baseline findings.

The mothers who tested p24 HIV positive were referred for confirmation using PCR or ELISA. Without the extra tests, it was not easier to directly link those mothers to care. There is a need in the national guidelines to provide provision to link person(s) who tested negative using the national HIV algorithm (12).

## Data Availability

The original contributions presented in the study are included in the article/supplementary material, further inquiries can be directed to the corresponding author.
